# Sequential extraction procedure to obtain the composition of terrigenous detritus in marine sediments

**DOI:** 10.1016/j.mex.2020.100888

**Published:** 2020-04-20

**Authors:** Margit H. Simon, Daniel P. Babin, Steven L. Goldstein, Merry Yue Cai, Tanzhuo Liu, Xibin Han, Anne A. Haws, Matthew Johns, Caroline Lear, Sidney R. Hemming

**Affiliations:** a NORCE Norwegian Research Centre, Bjerknes Centre for Climate Research, Bergen, Norway; b SFF Centre for Early Sapiens Behaviour (SapienCE), University of Bergen, Post Box 7805, 5020, Bergen, Norway; c Lamont-Doherty Earth Observatory of Columbia University, 61 Rt 9W, Palisades, New York 10964-8000, USA; d Department of Earth and Environmental Sciences, Columbia University, New York NY USA; e Key Laboratory of Submarine Geosciences, SOA Second Institute of Oceanography, Hangzhou, China; f Department of Earth and Environmental Sciences, Boston College, Chestnut Hill, MA 02467, USA; g School of Earth and Ocean Sciences, Cardiff University, Park Pl, Cardiff CF10 3AT UK

**Keywords:** Cation exchange wash, Sediment leaching protocol, Composition of detrital fraction

## Abstract

The geochemical and isotopic composition of terrigenous clays from marine sediments can provide important information on the sources and pathways of sediments. In order to extract the detrital signal from bulk marine sediments, standard sediment leaching methods are commonly applied to remove carbonate and ferromanganese oxides. In comparison to most previous studies that aimed to extract the terrestrial signal from marine sediments we additionally applied a CsCl wash throughout the sample preparation Simon et al. [1]. The motivation behind that extra step, not frequently applied, is to remove ions that are gained on the clay surface due to re-adsorption of authigenic trace metals in the ocean or during the leaching procedure and thus could alter the original composition of the detrital fraction if no cation exchange was applied. Here we present an improved and detailed step-by-step leaching protocol for the extraction of the detrital fraction of bulk deep-sea sediments including commonly used buffered acetic acid and acid-reductive mix solutions including a final cation exchange wash.

• standard method to remove carbonate and ferromanganese oxides and Stokes settling to isolate the clay fractions
• additional application of cation cation exchange wash (CsCl)
• removal of ions that are gained on the clay surface due to adsorption of authigenic trace metals in the ocean or during the leaching procedure

## Graphical Abstract

**Figure:**
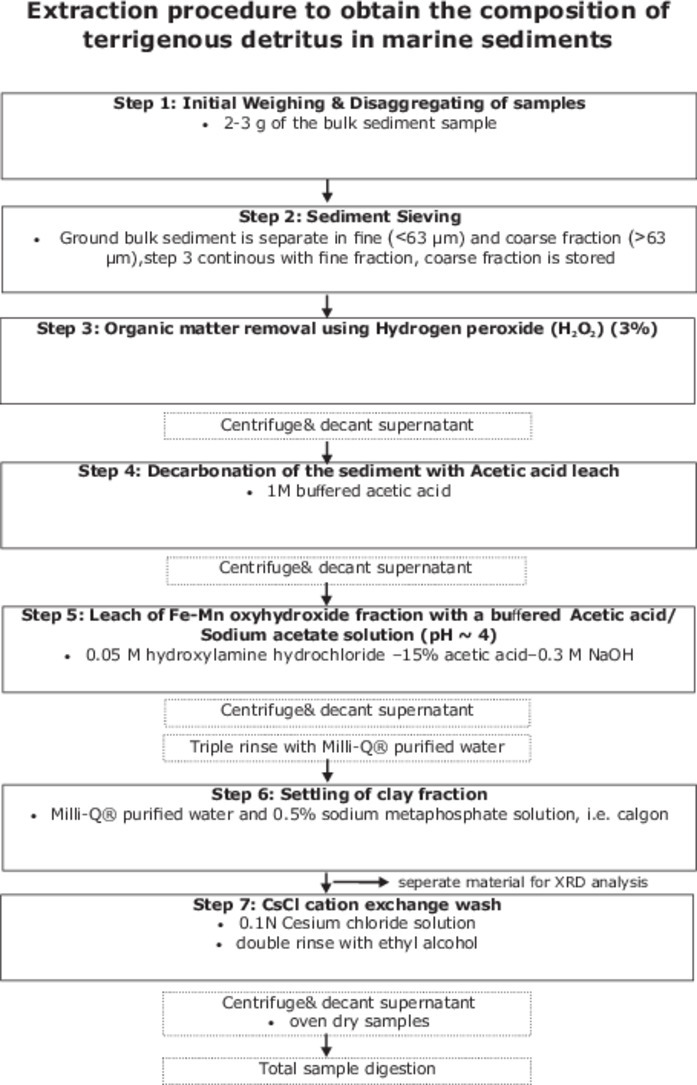

**Specifications table**

**Table:** 

Subject Area	*• Earth and Planetary Sciences*
Method name:	*Sediment leaching protocol including a cation exchange wash*
Name and reference of original method	*The following method is a sediment leaching procedure for marine sediments that can be applied to approximately a batch of 10–20 samples at the time. Procedures in part modified after*[2]*.*

## Method details

**Fig. 1:**
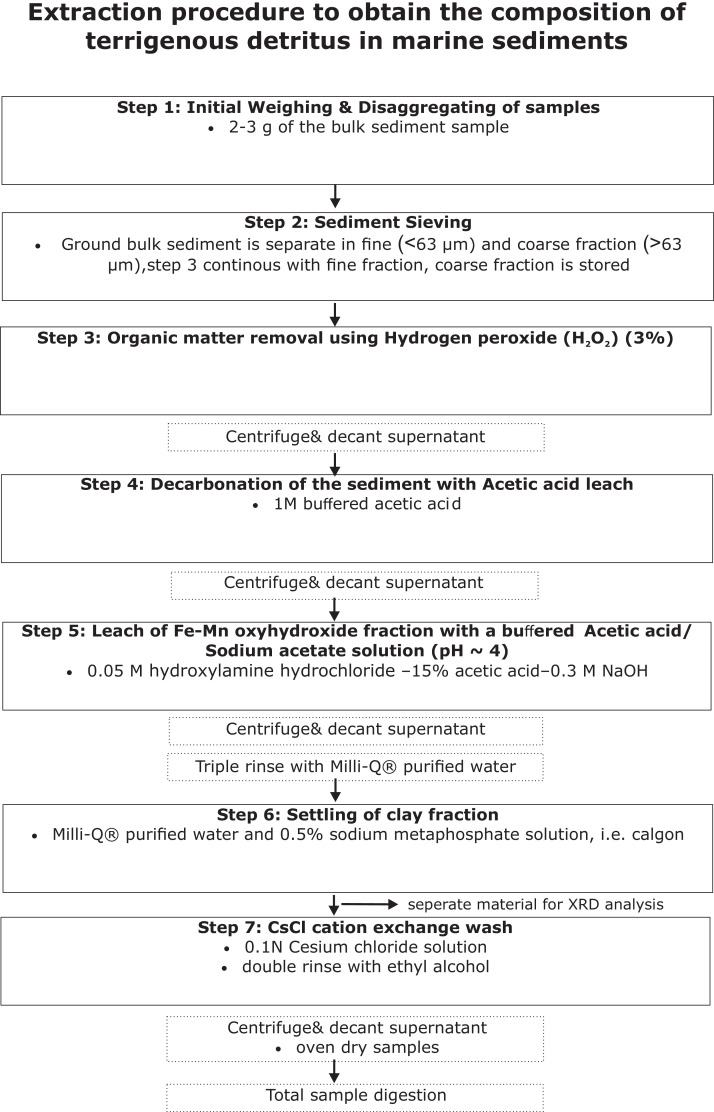
Schematic view of the applied sediment treatments (leaches, rinses, total digestions) and their order.

Following is the outline and detailed protocol (Fig. 1) for the preparation of equipment, labware, reagents and solution used in the protocol for sediment sample leaching in order to extract the detrital signal from marine sediments. Because this is not a method of analysis, subsections normally found relating to risks of contaminations and quality control of the chemical analyses have been omitted. For more information on these subjects the reader is referred to the main publication [3] where these details are published and standard treatment is described.

### Hardware/glassware

Glassware and implements used in these procedures include the following:

- reagent bottles: 1000mL and 2000mL
- 400mL glass beaker
- 50mL centrifuge tube
- 15mL centrifuge tubes
- squirt bottle 250mL
- fine test sieve, pore size 63µm
- ph-indicator strips ph 0–14 universal indicator

### Chemicals

Chemicals used in this procedure include the following:

- Hydrogen peroxide 3% (H2O2, Fisher Chemical, Certified, Catalog No. H324-500)
- Sodium acetate (C2H3NaO2 Anhydrous, Fisher Chemical, Certified ACS, F.W. = 82.03, S210-2)
- Glacial acetic acid (CH3COOH, Fisher Chemical, Certified ACS, F.W.=60.05, product A38C-212)
- Hydroxylamine hydrochloride (H3NO•HCl, Alfa Aesar, 99% purity, F.W. 69.49, Stock #: A15398)
- Sodium hydroxide pellets (NaOH, Fisher Chemical, Certified ACS, F.W. = 40, Catalogue #: S318-1)
- 0.5% sodium metaphosphate solution ((NaPO3)6, Fischer Chemical, Laboratory Grade Powder, Catalogue #: S333-500)
- Cesium chloride (CsCl, INDOFINE, Research Grade, F.W. = 168.36, Product #: MB1006)
- Ethanol (C2H6O, Ethyl Alcohol Denatured (Proprietary Solvent), Certified, Fisher Chemical, F.W. = 46.069, Catalogue #: A407-500)
- deionized water (Milli-Q® purified water that has resistance of 18.2 MΩ•cm at 25°C)

### Solutions

Preparation of 2L of of buffered acetic acid solution (Step 4):

1. Begin with 1L MQ water in a 2L bottle (MQ water = Milli-Q® purified water that has resistance of 18.2 MΩ•cm at 25°C)
2. Add 164g sodium acetate (C2H3NaO2 Anhydrous, Fisher Chemical, Certified ACS, F.W. = 82.03, S210-2)
3. Add 114mL glacial acetic acid (CH3COOH, Fisher Chemical, Certified ACS, F.W.=60.05, product A38C-212)
4. Fill to 2L with MQ

Preparation of 500mL of buffered acetic acid/sodium acetate (pH 4) solution (Step 5):

1. start with 250mL MQ
2. Add 1.84g hydroxylamine hydrochloride (H3NO•HCl, Alfa Aesar, 99% purity, F.W. 69.49, Stock #: A15398)
3. Add 76mL glacial acetic acid (CH3COOH)
4. Add 10g NaOH pellets (Fisher Chemical, Certified ACS, F.W. = 40, Catalogue #: S318-1)
5. Fill to 500mL with MQ
6. Check pH after 1hr; should be ~4

### Sieving, leaching, & settling procedure

**Step 1: Initial Weighing & Disaggregating of Samples**

1. Weigh 2-3g of the sample to be sieved in a dry, tared 50mL centrifuge tube and record the mass.
2. Also weigh a clean, labeled 63µm sieve for each sample and record the mass.
3. Fill each tube to ~30mL with deionized water to disaggregate the samples. Disaggregating may be facilitated by vortexing and sonicating (the better the samples are disaggregated, the less water will be necessary to sieve the samples, thus saving time later).

**Step 2: Sediment Sieving**

1. Place a sieve in a 400mL beaker with a corresponding label, and pour the sample onto the sieve; use a squirt bottle with deionized water to ensure the whole sample is transferred.
2. Use the spray bottle to wash the fine fraction through the sieve. This is complete when the water coming out of the sieve is clear and the coarse fraction does not appear to contain any clumps when viewed under a microscope.
3. Place the sieves with the coarse fraction in the oven at 50°C, and weigh them when dry. Transfer the coarse fractions to small glass vials for storage. This is best done by emptying the sieve onto creased weighing paper and using the weighing paper to pour the coarse fraction into the vial.
4. Allow the fine fractions to settle in the beakers overnight; if they are still cloudy the next day (or longer if needed), a few drops of buffered acetic acid (recipe see Step 4 above) may be added to facilitate settling.
5. After the fine fractions have settled, remove as much water as possible and recombine multiple beakers if necessary.
6. Return the fine fraction to the original centrifuge tube; it may be necessary to fill the tubes to 50mL, centrifuge for 30min at 2400rpm, decant the water, add the rest of the water and fine fraction, and centrifuge and decant again.

**Step 3: Organic matter removal using Hydrogen peroxide (H_2_O_2_) (3%)**

1. Place a sample in each beaker and add about 50-100mLof dilute hydrogen peroxide (H_2_O_2_). Stir each beaker with the glass rod to suspend the sample. Rinse the glass rod between each sample and dry with a lab tissue. When bubbling stops or slows, add another 50-100mL of dilute hydrogen peroxide (H_2_O_2_) and restir the suspension.

*NOTE: This step can also be performed at higher temperature (60–70°C ), with your beaker in a hot water bath. Moreover the hydrogen peroxide concentration can be increased to e.g. 6% if OM concentration is high.*

2. When the addition of hydrogen peroxide to the samples no longer causes bubbling, the organics have been removed. Allow the suspension to settle and carefully siphon or pour off the supernatant liquid, or wash the sample by centrifuging.

*NOTE: It should be considered that in some cases, depending on the sediment properties, an organic oxidative leaching step with hydrogen peroxide (3%) would still not be sufficient to remove various organic phases. Stronger treatments for OM removal can however attack the clays and leach away soluble elements including the REE that would affect the trace element measurements in the end. Hence this step might have to be adapted individually according to the setting.*

**Step 4: Decarbonation of the sediment with Acetic acid Leach** (Adapted from [2])

1. Vortex the samples to disaggregate them a bit, and fill the tube to ~30mL with the 1M buffered acetic acid. Liberate any CO_2_ produced.
2. Vortex to disaggregate completely.
3. Release CO_2_ again and ensure that the caps are tight before placing on the rocking table for 6-12hr, or overnight.
4. Centrifuge for 30min at 2400rpm, and decant the clear acid solution to waste.
5. Repeat Steps 1–4 until the sediment no longer appears to react with the acid; there will be no more carbon dioxide formed, and no bubbles will form when the acid is added (however, bubbles may form as the sediment shifts when the acid is poured over it, and the acid itself may foam when vortexed, even if no reaction is taking place).

**Step 5: Leach of Fe-Mn oxyhydroxide fraction with a buffered Acetic acid/ Sodium acetate solution (pH ~ 4)**

1. Fill each tube to ~20mL with buffered acetic acid/ sodium acetate solution and place on rocking table for at least 8hr or overnight.
2. Centrifuge for 30min at 2400rpm, and decant clear solution.
3. If the supernatant was colorless, only one leach is necessary. If it is tinted, repeat until the supernatant is clear.
4. Fill the tubes to ~30mL with MQ water, vortex to disaggregate, centrifuge at 2400rpm for 30 min, and decant.
5. Repeat step 5 two additional times. As the acid is removed, settling may become more difficult, so centrifuging at a higher speed and for a longer time may be necessary.

*INFO: This step deals with the leaching of Fe-Mn oxides fraction with the acetic acid/sodium acetate buffer (pH 4). The acetic acid (CH3COOH) reacts with sodium (N*a*OH) to produce the sodium acetate (CH3COONa). The two compounds are in contact with hydroxylamine hydrochloride (NH2OH, HCl) in order to buffer the solution at pH 4.*

*NOTE: This sequence of procedures deviates from that of Gutjahr et al.* [2] *who followed the initial carbonate leaching with a cation exchange step with MgCl_2_ in order to desorb cations prior to leaching the dispersed Fe-Mn oxy-hydroxide for authigenic Nd isotope measurements. Moreover, we did not use the complexing reagent EDTA as originally suggested in the protocol during the leaching of the authigenic Fe-Mn oxyhydroxide fraction. We recommend the application of a sequence including EDTA (HH–acetic acid–Na–EDTA) following Gutjahr et al.* [2]*.*

**Step 6: Settling of clay fraction**

Separations of grains smaller than 20 microns are carried out by settling in a column of water in glass cylinders. Particles will settle in the water according to Stokes Law:

V = 2/9(ρg –ρf) g r^2^/η
where:
V = the settling velocity in cm/sec
ρg = density of the mineral grains (2.6 - 2.8 g/cm^3^ for clay minerals)
ρf = density of fluid (1g/cm^3^ for water)
g = acceleration due to gravity (980 cm/sec^2^)
r = radius of the mineral particle (10^−4^ cm for clays)
η = viscosity of water (10^−2^ g cm/sec2)

1. Label as many clean, 100mL graduated cylinders as you have samples with a piece of tape placed 5cm down from the 100mL mark. Also label a set of clean centrifuge tubes with the sample names and size fraction (for our purposes, <2µm).
2. Add ~30mL MQ water and ~2mL 0.5% sodium metaphosphate solution, i.e calgon ((NaPO3)6, Fischer Chemical, Laboratory Grade Powder, Catalogue #: S333-500) to each tube after decanting the 3rd MQ rinse. Sodium metaphosphate, a detergent, helps disperse the clays and prevent flocculation.
3. Vortex until samples are disaggregated.
4. Pour the contents of each tube into the appropriate cylinder, using the MQ squirt bottle to make sure that all sediment is transferred (and save the original tube for the 2-63µm fraction).
5. Fill the cylinders to the 100mL mark with MQ water and cover with a square of parafilm.
6. Sonicate the cylinders for about 5 min, then shake and invert them a few times to evenly disperse the sediment throughout the cylinder. At this point, it is extremely important to make sure the sample is fully disaggregated. If heavy clumping is observed, sonicate and shake the cylinder until the clumps disappear - this may take some effort and multiple sonicating rounds.
7. Place the cylinders in a safe place on the counter where they won't be bumped or otherwise agitated, and start a timer for 3hr 52min. Be sure to leave enough room between the cylinders so you can reach around them a little more easily later. Staggering them is also a good idea.
8. After time is up, use a turkey baster to transfer the top 5cm of water (25mL) to the clean centrifuge tubes labelled earlier. Clods (for Ar) may be made at this point if desired.
9. To reset the cylinders for another round of settling, add ~2mL calgon (for one or two settling rounds only) to each and fill to 100mL with MQ and cover with parafilm, sonicate for about five minutes, shake and invert, and reset the timer. Only add more calgon prior to the second round of settling.
10. Centrifuge the tubes at 4000rpm for 30min to settle the <2µm fraction. The resulting supernatant should be translucent, and it will likely be tinted. If the solution is still cloudy when removed from the centrifuge, return it to the centrifuge for a longer length of time.
11. Repeat Steps 8–10 until settling is complete or until enough 2µm fraction has been collected to perform desired analyses. If bringing settling to completion, a good way to tell if it is complete is to hold up a paper with black and white type on it. The black and white should contrast sharply and the letters shouldn't look clouded.
12. Transfer the 2-63µm fraction to the original centrifuge tubes (if settling is complete or close to complete, allowing the remaining sediment to settle for a while and then pipetting clear water off will save time when transferring). Use the MQ squirt bottle to ensure all sediment is transferred. This can be done by pouring the sediment directly into the tubes from the cylinders, and then centrifuging for 30min, decanting, and continuing to add to the tubes until the cylinder is empty. Alternatively, if you are in a hurry and need the cylinders immediately, transferring the sediment to a beaker first and then the tubes (following the same procedure) is also an option. Additionally, if settling was stopped after a particular number of rounds and was not fully completed, be sure to note on the tubes that there is still some <2µm fraction present).

**Step 7: CsCl Cation Exchange Wash** (Adapted from [3])

1. Fill each 50mL centrifuge tube up to the 15mL line with MQ water.
2. Label new 15mL centrifuge tubes with the sample name + “w/ CsCl”
3. Vortex to completely disaggregate sample.
4. Quickly, while sample is still completely suspended, pipette 5mL of sample from the 50mL tube to the new 15mL tube.
5. Using the same tip, pipette 5mL of MQ water into the 15mL centrifuge tube twice to clear any remaining sample from the pipette tip.
6. Pipette 5mL of MQ 3 times into waste to wash the pipette tip and avoid contamination.
7. Obtain fresh MQ water and repeat Steps 3–6 for the remaining sample.
8. Centrifuge 15mL tubes for 90 min at 4000rpm, and remove the water with a pipet. Do not pour.
9. Pipet 10mL 0.1N Cesium chloride (CsCl, INDOFINE, Research Grade, F.W. = 168.36, Product #: MB1006) solution into each tube.
10. Vortex each sample to completely disaggregate and suspend it; this will be difficult and sonicating may be necessary.
11. Rotate the tubes for 24 hours to ensure complete interactions between the sediment and the reagent.
12. Centrifuge samples at 2000rpm for 20min; the Cs ions will help the clay settle much more easily.
13. Decant each sample; you should be able to pour without losing any sample.
14. Add 10mL MQ, vortex until suspended, centrifuge at 2000 RPM for 20 min, and decant. Add 10mL ethanol (C2H6O, Ethyl Alcohol Denatured (Proprietary Solvent), Certified, Fisher Chemical, F.W. = 46.069, Catalogue #: A407-500), vortex, centrifuge, and decant as above; repeat this step a second time.
15. Let sample dry in the oven at 50°C. These samples cannot be freeze dried due to low freezing point of ethanol.

## Declaration of Competing Interest

The authors declare that they have no known competing financial interests or personal relationships that could have appeared to influence the work reported in this paper.
